# Strategies for the Fortification of Human Milk in Preterm Infants: A Systematic Review

**DOI:** 10.7759/cureus.73380

**Published:** 2024-11-10

**Authors:** Francisco Contreras Chova, Andrea Villanueva-García, JL González-Boyero, Ana M Campos-Martínez, Enrique Blanca-Jover, Antonio E Jerez-Calero, José Uberos-Fernández

**Affiliations:** 1 Pediatrics and Neonatology, Hospital Universitario San Cecilio, Granada, ESP

**Keywords:** breastmilk fortification, donor human milk fortification, human milk, human milk fortifiers, preterm newborns

## Abstract

Breast milk is the best source of nourishment for both full-term and preterm newborns. However, in preterm newborns, exclusive breastfeeding can lead to nutritional deficiencies, with short- and long-term consequences on growth and neurocognitive development. Breast milk fortification is a widely used strategy to provide an adequate nutritional profile to these patients. There is currently a great heterogeneity in terms of fortification patterns and the type of fortifier. The present systematic review summarizes the current scientific evidence to evaluate the different strategies for fortification of human milk in preterm infants, their possible differences on benefits, and the hypothetical impact on prematurity outcomes.

## Introduction and background

Currently, the number of preterm births worldwide is estimated at around 13.5 million per year, which is approximately 10% of all newborns [1]. Prematurity is one of the main health problems in childhood, both in terms of mortality and subsequent health problems secondary to preterm birth [2]. The higher survival rate in recent years [3] poses a challenge in terms of optimizing therapeutic measures to minimize the impact of sequelae secondary to preterm birth. In this sense, the optimization of nutritional measures can play a fundamental role. In fact, prematurity is considered a nutritional emergency, given the evidence that shows a delay in the acquisition of optimal extrauterine growth parameters due to nutritional deficiency in this group of newborns [4].

Breast milk (BM) as the best source of nutrition for both term and preterm newborns (PTN) is actually unanimously accepted due to its short- and long-term benefits [5-7]. Thus, in PTN, BM has been shown to reduce the incidence of necrotizing enterocolitis (NEC) and infections, and, therefore, mortality in PTN [8]. However, from a nutritional point of view, BM does not provide the necessary nutrients to PTNs, especially those <1,500 grams of birthweight, fed at standard volumes of 150-180 milliliter per kilogram [7,9,10], considering intrauterine growth levels as objectives [11] (Table 1 [12-14] and Table 2 [15-21]). Feeding with donated BM (usually mature milk), provided through milk banks and subjected to a pasteurization process, confers part of the benefits provided by the mother's own milk in terms of improving gastric tolerance, reducing NEC, or cardiovascular benefits in the long term, but precisely the pasteurization method alters the biological and nutritional/endocrine properties of BM [22].

**Table 1:** Evolution of the nutritional properties of breast milk in the first weeks after birth and their comparison with the nutritional requirements of the PTN PTN, preterm newborns; TN, term newborns

	Nutritional recommendations for PTN <1,500 g (12-14)	Estimated nutritional values of breast milk for TN (100 mL)			Estimated nutritional values of breast milk for PTN (100 mL)		
		1st week	2nd week	Week 3	1st week	2nd week	Week 3
Energy	110-130 kcal/kg	60 (44-77)	67 (47-86)	66 (48-85)	60 (45-75)	71 (49-94)	77 (61-92)
Proteins	3,5-4.5 g/kg	1.8 (0.4-3.2)	1.3 (0.8-1.8)	0.9 (0.6-1.2)	2.2 (0.3-4.1)	1.5 (0.8-2.3)	1.4 (0.6-2.2)
Fat	4,5-8,1 g/kg	2.2 (0.7-3.79	3.0 (1.2-4.8)	3.4 (0.6-5.2)	2.6 (0.5-4.7)	3.5 (1.2-5.7)	3.5 (0.8-6.5)
Carbohydrates	11-13 g/kg	7-8	7-8	7-8	7-8	7-8	7-8
Calcium (mg)	150-220 mg/kg	26	28	27	26	25	25
Phosphorus (mg)	75-140 mg/kg	12	17	16	11	15	14
Iron	1-3 mg/kg	0.1	0.05	0.04	0.1	0.1	0.1

**Table 2:** Comparison of nutritional requirements in PTN <1,500 g with the estimated supply of mature breast milk of term newborn (TN), PTN, and HM with complete fortification *Estimation of upper and lower limits of the main commercial brands, according to the manufacturer's specifications. It includes fortifiers of bovine origin and breast milk derivatives **mg/dL PTN, preterm newborns; TN, term newborns; HM, human milk

	Nutritional values required in PTN <1,500 g (100 mL)	Estimated composition of mature breast milk for TN (100 mL)	Estimated composition of mature breast milk for PTN (100 mL)	Estimated composition of HM + full fortification (100 mL)*
Energy (kcal)	110-130	68	66	82-97
Proteins(g/k)	3.5-4.5	0.9	1	2-3
Fat (g/k)	4.5-8	3.4	3.7	3 - 5.4
Calcium (mg)	150-220 mg/kg	26**	29**	117-140**
Phosphorus (mg)	75-140 mg/kg	16**	12**	63-77**
Iron (mg/100 mL)	1-3 mg/kg	0.05	0.1	

Breast milk fortifiers

Breast milk fortifiers were developed and commercially introduced in the 1980s [7], and their use has been progressively extended to most neonatal intensive care units, in parallel with the achievement of better morbidity and mortality figures for PTN.

Common multicomponent and single-component fortifiers contain varying amounts of protein, energy, minerals, trace elements, vitamins, and electrolytes [7], with the aim of achieving growth levels similar to those obtained under standard intrauterine conditions [23].

Types of Fortifiers According to Composition and Characteristics

Origin: the most common fortifiers are multicomponent preparations of bovine origin, with fortifiers based on pasteurized human milk [7,24] or from another origin [25-27], and there are also single-component preparations of proteins, carbohydrates, or fats [7].

Presentation: liquid versus powder presentation

Protein content: fortifiers providing moderate protein content (addition of 1.1 to 2.2 grams of protein/100 mL of final volume) versus fortifiers that provide high protein content (>1.4/100 mL of final volume)

Protein structure: proteins not treated versus hydrolyzed proteins

Acidified versus non-acidified: only applicable to liquid fortifiers [28]

Types of Fortification

There are two fundamental types of fortification, standard and individualized, which, in turn, can be classified as adjusted or targeted [29].

Standard fortification consists of adding a fixed amount of multi-component fortifier to achieve a predetermined proportion in 100 ml of milk. In adjusted individualized fortification, on the basis of standard fortification according to the above criteria, once the complete or tolerated level of fortification has been reached, protein intake is regulated according to the patient's own metabolic response, by measuring blood urea nitrogen (BUN) levels [7], and adding a single-component protein module, if necessary. This is intended to prevent both overfeeding and protein deficiency [30]. Targeted fortification of human milk is based on its composition, through analysis at the patient's bedside, and the nutritional recommendations for gestational and postnatal age [7,31], providing the necessary nutritional components (macronutrients) in a targeted way.

The aim of this systematic review (SR) is to analyze the current evidence on the best fortification strategies in relation to the somatometric evolution of preterm infants, neurodevelopment, aspects related to the possible correlation between types of fortification, and the incidence of comorbidities typical of PTN such as NEC and sepsis.

## Review

Material and methods

This SR aims to review and establish best strategies for the fortification of BM, both mother's own milk and donor milk (DM), in the nutrition of the PTN.

Search Strategy

Bibliographic search was performed for articles published between January 2019 and December 2023 based on clinical trials (CTs), randomized clinical trials (RCTs), meta-analyses (MAs), and SRs that included preterm or low birthweight infants as study subjects. Other inclusion criteria were as follows: a) studies focusing on the assessment of various BM fortification strategies and b) comparisons between different intervention groups in this population.

Sources of Information

Search was carried out on the following database websites: PubMed, Scopus, and the Cochrane Library. The mesh terms used in the search are summarized in Table 3.

**Table 3:** Mesh terms used per database website

Database	Terms Used
PubMed	“Human milk fortification” [Mesh] AND “premature” [Mesh] “Human milk fortifier” [Mesh] AND “preterm” [Mesh] “Human milk fortifier” [Mesh] and “premature” [Mesh] “Breast milk supplementation”[Mesh] AND “ preterm” [Mesh] “Hydrolyzed fortification” [Mesh] AND “ preterm” [Mesh] “Breast milk” [Mesh] AND “fortification” [Mesh] AND “very-low-birth-weight” [mesh] “Breast milk” [Mesh] AND “fortification” [Mesh] AND “donated milk” [Mesh] AND “very-low-birth-weight” [Mesh] “Human milk” [Mesh] AND “fortification” [Mesh] AND “preterm formula” [Mesh] AND “very-low-birth-weight” [Mesh]
Scopus	“Breast milk” [Mesh] AND “fortification” [Mesh] AND “premature” [Mesh] “Breast milk supplementation” [Mesh] AND “ premature” [Mesh]
Cochrane	“Human milk fortifier” [Mesh] AND “preterm” [Mesh] “Human milk fortification” [Mesh] AND "Breast milk supplementation” [Mesh] AND “very-low-birth-weight” [Mesh]

The PRISMA 2020 search list was used for SRs [32]. The PICO question was designed as follows:

P (population): preterm infants

I (intervention) and C (control): fortification of BM in its various modalities

O (results): somatometry, fecal microbiota analysis, neurological development, osmolarity, cost analysis

S (study design): review of CTs, RCTs, MAs, and SRs

Initially, all articles obtained in searches with the above criteria in that period of time were considered for inclusion for evaluation.

Exclusion criteria were as follows: a) articles not written in English or Spanish, (b) non-human studies, (c) articles that do not specifically differentiate preterm or low birthweight for gestational age, (d) articles with insufficient or confusing information, e) non-randomized studies, and f) studies in preterm subjects but focused on specific pathologies.

Study Selection and Data Extraction Process

**Figure 1:**
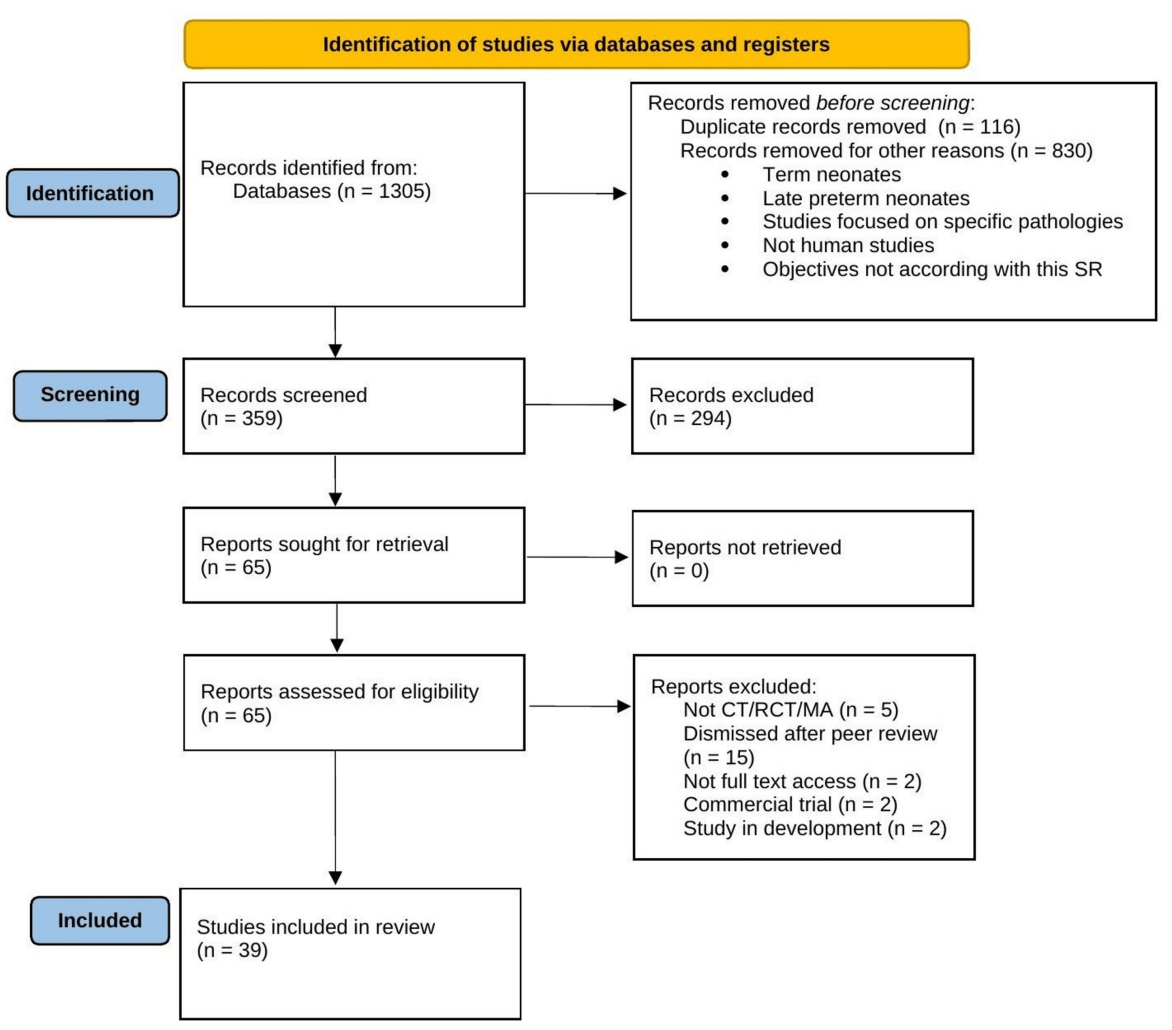
PRISMA 2020 flow diagram of the studies selected for analysis PRISMA, Preferred Reporting Items for Systematic Reviews and Meta-Analyses

The initial exclusion criteria set out were applied to the results of the initial search with the terminology mentioned above, obtaining 359 references. Once screened for exclusion criteria and duplicate references removed, articles obtained (65) were reviewed independently by two reviewers (F.C.C. and A.V.G.) for final eligibility screening. In case of disagreement, both reviewers submit the articles to a process of discussion until they are included or definitively excluded (Figure 1) [33]. On definitively selected articles, results were collected if they are statistically significant and relevant in terms of results and if they were included in the objectives of this review, regardless of whether or not they constitute the primary objective of the source studies.

Fundamental data extracted from the articles selected for further analysis included the following: identification of the article (author(s), title, publication, year), type of study, specific characteristics of each study (duration of the intervention, initiation of the intervention, type of milk, etc.), and characteristics of the sample population included in each study.

Studies were categorized according to their quality using the Oxford Standards of Evidence criteria [34].

Results

Study Characteristics

A total of 39 articles are selected for final review. Of these, 27 were RCTs/CTs and 12 were SRs and/or MAs. They are detailed with their characteristics summarized in Table 4 [35-72].

**Table 4:** Characteristics of the studies included in the systematic review BF, breastfeeding; BW, birthweight; CT, clinical trial; GA, gestational age; GI, gastrointestinal; HC, head circumference; HM, human milk; MA, meta-analysis; MRI, magnetic resonance imaging; NEC, necrotizing enterocolitis; PTN, preterm newborn; RCT, randomized controlled trial; SR, systematic review; VLBW, very low birthweight

Authors	Article type	Sample (n)	Sample characteristics	Intervention and relevant conclusions
Embleton et al. (2023) [35]	RCT	126	GA<30	Analysis of the microbiota of premature infants fed with breast milk and fortifier derived from human milk versus those fed with breast milk and fortifier of bovine origin. There are no differences in gut bacterial diversity, but differences in bacterial species. There were no relevant differences in terms of key neonatal morbidities, weight gain, or length of hospital stay.
Hemmati and Ghassemzadeh (2023) [36]	RCT	77	GA≤33 and BW<1,500 g	Protein supplementation (4-5 g/kg/day) significantly improves anthropometric parameters. Protein supplementation can complement routine feeding protocols for infants with VLBW without any short-term adverse effects.
Reis et al. (2023) [37]	RCT	114	GA<29 or GA<34 plus LBW	Individualized + optimized nutrition (experimental group) vs only optimized nutrition. Comparison of neurocognitive development. The type of HM supplement had no significant effect on Bayley scores assessed between 18 and 38 months.
Salas et al. (2023) [38]	RCT	150	GA<28	Unfortified BM feeding vs early fortification feeding derived from HM. In PTN, early fortification does not increase the accumulation of lean mass at 36 weeks’ postmenstrual age, but it can increase length and reduce HC.
Seliga-Siwecka et al. (2023) [39]	RCT	75	GA<32	Standard fortification vs directed fortification. No significant differences in weight gain, length, or HC. Study interrupted by cases of severe intolerance.
Asbury et al. (2022) [40]	RCT	119	BW<1,250 g	Breast milk with fortifiers based on human vs bovine milk-based fortifiers. Infants fed HM-derived fortifiers show lower microbial diversity, more uniform microbial profiles characterized by a higher abundance of unclassified Proteobacteria and Enterobacteriaceae, and lower abundance of Firmicutes and Clostridium sensu stricto compared to infants fed fortifiers derived from bovine milk. Dose-dependent relationship between individual components of enteral feeding, such as type of HM (breast vs donor) and milk volumes and the microbiota.
Hamidi et al. (2022) [41]	RCT	30	GA<32 and BW<1,250 g	Protein supplementation at high doses (4.8 g/kg/day) is associated with better weight growth (increase in HC and weight) and development of the immune system. Lower incidence of sepsis in high-dose protein supplements.
Hilditch et al. (2022) [42]	MT	378	GA<37	Early fortification is not associated with significant improvements in growth or differences in adverse enteral outcomes with delayed fortification.
Klamer et al. (2022)[43]	RCT	214	GA<32	PTN fed up to 4 months of age with BF had better scores on the verbal comprehension index (WISC-IV scale) and motor skills compared to children fed artificial formula, even in analyses adjusted for confounding factors. There was no difference between fortified and unfortified breast milk.
Kumbhare et al. (2022) [44]	RCT	30	GA 26-30	Type of HM (breast vs donor) is an important determinant of PTN microbiome development, while fortifier origin (human vs bovine) has minimal impact.
Masoli et al. (2022) [45]	RCT	158	BW 500-1,500 g	Powder vs liquid fortification. No differences in somatometrics between the two groups. Increased incidence of death from NEC in the powdered fortifier group, although not statistically significant.
Moreira-Monteagudo et al. (2022) [46]	SR and MA	2,524	GA<36 and/or BW<1,500 g	Systematic review with somatometric and neurodevelopmental objectives between different forms of preterm infant feeding. Gut microbiota analysis as a secondary objective.
Nogueira-Pileggi et al. (2022) [47]	RCT	40	BW 750-1,500 g	Fortification of breast milk with lyophilized concentrate from HM vs standard fortification (bovine origin). No somatometric differences between the two groups. No significant differences in safety analyses (NEC, sepsis, gastrointestinal intolerance). Lyophilized fortification from donor HM was considered safe and tolerable for use in hemodynamically stable patients with VLBW.
Salas et al. (2022) [48]	RCT	56	GA 25-28	High-protein fortification vs fortification with usual protein levels. Higher percentage of fat-free mass and better length and weight values in the fortified group with high protein levels.
Uthaya et al. (2022) [49]	RCT	38	GA<30	Analysis of body composition (measured by MRI) in preterm infants with supplementation derived from HM vs supplementation based on cow's milk. No statistically significant differences.
Bridges et al. (2021) [50]	SR and MA	591	GA 25-31	Very low-quality evidence in favor of greater linear growth in PTN fed liquid HM fortifier, as well as greater weight gain in those fed casein hydrolyzed fortified HM, compared to control (powdered fortifiers). Insufficient evidence to recommend liquid HM fortifier regarding NEC or late sepsis.
Chinnappan et al. (2021) [51]	RCT	123	GA<34	Powder fortification of artificial formula for preterm infants is not inferior to supplementation with fortifiers derived from HM. Preterm formula may be a good option for fortifying the milk of preterm infants in resource-limited countries.
Grace et al. (2021) [52]	SR and MA	332	GA<34 and/or BW<1,500 g	It is suggested that incidence of NEC was lower in the group with HM-derived fortification vs cow-derived fortification group. There were no differences in sepsis. The evidence from the study was low due to the lack of blinding in the study.
Kumar et al. (2021) [53]	SR + MA	423	PTN	Suggests with low evidence that artificial formula fortification of breast milk is superior to non-fortification and may be a safe alternative to bovine fortifiers, especially in resource-limited countries.
Rochow et al. (2021) [54]	RCT	427	GA<30	Standard vs test-adjusted supplementation of breast milk; evidence that individualized fortification of breast milk improves nutrition and growth quality.
Alyahya et al. (2020)[55]	SR	N.E.	BW<1,500 g	Early fortification vs late fortification; not enough evidence to define the optimal time to start fortification.
Ananthan et al. (2020) [56]	SR and MA	389	GA	Fortification based on HM vs bovine origin. Fortification based on HM is associated with a lower risk of NEC, with low-quality evidence, as well as less weight gain.
Amissah et al. (2020) [57]	SR and MA	204.	GA<36	Standard fortification vs fortification with extra protein content. Short-term growth enhancement in preterm infants supplemented with high protein levels. No applicable evidence on length of hospital stay, prevalence of NEC, or GI intolerance.
Basu et al. (2020) [58]	SR and MA	309	BW<2,000	Early fortification vs late fortification very low-quality evidence. There is no significant difference in the growth parameters of preterm infants. Early fortification showed a significant increase in hospital stay and a non-significant increase in food intolerance and NEC.
Brion et al. (2020) [59]	RCT	120	GA <29 or <35 and small for GA	Comparison between individualized, adjusted (optimized) and targeted fortification. There was no improvement in weight gain, linear growth, or weight/length proportion between both procedures.
Bulut et al. (2020) [60]	RCT	49	GA<32	Individualized, adjusted vs targeted fortification. Targeted individualized protein fortification had more beneficial effects on short-term growth compared to adjusted fortification method.
Fabrizio et al. (2020) [61]	SR and MA	521	GA<37 or BW<2,500 g	Individualized fortification adjusted vs standard. Moderate- to low-certainty evidence suggests that individualized fortification increases growth rates of weight, length, and HC compared to standard fortification. Low evidence for out-of-hospital outcomes after discharge.
Gao et al. (2020) [62]	SR and MA	861	GA<37	High-protein fortification vs standard content. Statistically significant, higher weight gain among preterm infants fed high protein fortification. No evidence on relationship between protein concentration and other clinical problems such as sepsis, NEC, or days to full enteral feeding.
Quan et al. (2020) [63]	RCT	51	GA<34 n	Individualized fortification vs standard fortification. Better rate of weight gain in the individualized fortification group than the standard group.
Parat et al. (2020) [64]	RCT	36	BW<1,500 g	Individualized fortification aimed at protein intake vs standard. Targeted fortification increases protein intake and provides better fat-free mass figures, which is associated with better neurological development in PTN.
Thanigainathan and Abiramalatha (2020) [65]	SR and MA	237	BW<1,500 g	Early fortification vs late fortification. Early fortification has little or no effect on growth outcomes, with low evidence. Low or no effect on NEC incidence, time to reach full enteral nutrition, or extrauterine growth retardation at discharge incidence.
Agakidou et al. (2019) [66]	RCT	48	GA<32 and BW<1,500 g	Fortification of the mother's own breast milk standard vs oriented. Different fortification methods that provide different amounts of protein are associated with changes in IGF-1 and ghrelin levels, which can have an effect on neuroendocrine and metabolic programming. Protein intake was positively correlated with IGF-I levels and inversely correlated with ghrelin levels.
Adhisivam et al. (2019) [67]	RCT	80	GA<37	Fortification of pasteurized donor milk (safety analysis). No increase in incidence of NEC. Similar incidence of sepsis, mortality, length of hospital stay, or number of days to reach full enteral feeding.
Kadıoğlu et al. (2019) [68]	RCT	60	GA<32 and BW<1,500 g	Comparison of standard, directed, and adjusted fortification. Both adjusted and targeted fortification significantly improved median daily weight gain, HC, and length vs standard fortification.
Kashaki et al. (2019) [69]	CT	36	GA<1,500 g	Standard fortification vs standard fortification + protein addition. Differences in neurodevelopment at 3 years of age. No differences in HC at 3 years. Better neurological development in communication and gross motor skills areas, auditory, verbal language, cognitive, social connection, and motor skills (based on Newsha developmental Scale).
Premkumar et al. (2019) [70]	SR and MA	127	GA<37	Exclusive BF with HM-based fortifiers vs bovine. No differences between both in terms of NEC, mortality, intolerance, infections, or improvement in growth (low evidence level).
Toftlund et al. (2019)[71]	RCT	281	GA<32	Analysis of fortification after discharge (fortified breast milk, non-fortified breast milk, and premature formula) up to 4 months of corrected age. Premature infants fed with higher protein values (premature formula and fortified BF) had better lung function data.

Fortification Strategies and Their Implication on Somatometrics

Source of the fortifier: Finding the best fortification strategy to optimize the growth of PTNs is one of the challenges in neonatology. In this regard, the available literature does not show that the protein source (fortification of human vs bovine origin) influences growth and body composition. Uthaya et al. did not identify statistically significant differences in body composition (as assessed by MRI) in PTNs who received an exclusive diet of human milk equivalent in macronutrients compared to a diet containing cow's milk products [49]. Fortification of human milk with artificial formula is a potentially attractive strategy in developing countries, proving to be not inferior in terms of weight gain and incidence of extrauterine growth retardation at discharge, being a low-cost strategy [51]. However, despite the fact that its benefits and safety need further study, a recent meta-analysis suggests with low level of evidence that fortification of BM with infant formula may be a superior nutritional strategy to feeding with unfortified BM in terms of achieving somatometric parameters, with a similar profile of comorbidity development, and may be a safe alternative to bovine fortifiers for nutritional supplementation of PTNs in developing countries [53].

Fortification with high levels of protein versus standard levels: Protein is essential for neonatal development, both to support neurodevelopment and to achieve adequate weight gain [36]. The latest clinical guidelines recommend a daily protein intake of 3.5-4.5 g/kg in PTN [12]. However, there is no consensus on the optimal amount of protein content in fortifiers or on whether the use of higher amounts of protein represents a better alternative to traditional fortifiers in terms of growth support. In recent years, several studies have been conducted, including CTs to assess the implication in anthropometric growth and neurodevelopment in those PTNs who receive fortification with high protein intake (4-5 g/kg/day) compared to fortification with moderate protein intake (3.5-4 g/kg/day) [36,41,48,62,64,66].

Hemmati and Ghassemzadeh [36] and Hamidi et al. [41] conclude that high-dose protein supplementation significantly improves the growth of anthropometric parameters. Another RCT discusses the influence of high-protein supplementation and lean fat production [48]. This study indicates that in PTN fed with a higher intake of enteral proteins, the accumulation of fat-free mass increases and growth is promoted without causing an excessive accumulation of body fat, findings consistent with other studies that emphasize a higher protein intake and therefore a higher percentage of lean mass when the fortification modality is through individualization through the analysis of the composition of BM donated [64], which will be detailed below. Gao et al. in their SR assess the effect of protein supplementation and its implication in somatometry, as well as its relationship with possible adverse effects [62]. As in previous CTs [66], this review mentions that addition of proteins to human milk could increase serum IGF‐1 concentrations, thus producing a decrease in fat mass and improvement in the initial growth of preterm infants.

Fortification method: In the fortification method, there are currently two main strategies: standard and individualized, with the latter also divided into adjusted individualized and directed individualized, as previously mentioned [7,30,31]. In the last five years, several trials have compared these methods [54,59,60,63,68]. Kadioglu et al. found evidence of improved levels of weight, length, and head circumference in preterm infants who have received adjusted and targeted individualized fortification versus standard fortification, through improved caloric and protein intakes [68]. Rochow et al. compared individualized targeted fortification versus standard fortification, achieving in the intervention group better levels of macronutrient intake, weight gain, length and head circumference, as well as better percentages of lean mass [54]. Parat et al. also found better percentages of fat-free mass in the targeted versus standard fortification group, considering a higher quality of weight gain; regarding the measurement of skinfolds, only the measurement of the flank was statistically significant, while there were no differences in weight gain [64]. Quan et al. found better levels of weight gain in the first weeks of life in preterm infants fed with fortification adjusted according to the level of protein in BM, blood urea, and weight of the subject, compared to the control group [63]. On comparison between the two forms of individualized fortification, one trial found better results in all anthropometric parameters measured over a four-week period in favor of nutrition-oriented (milk composition analysis) [60]. On the other hand, the combination of both models of individualized fortification does not seem to provide better somatometric results in the short term [59]. A 2020 SR agreed on better outcomes during the intervention in favor of individualized fortification (in any of its variants) compared to standard fortification; however, it could not draw conclusions after the intervention period [61]. A more recent trial, on the other hand, found no statistically significant difference in the rate of weight gain during the period of supplementation in the targeted fortification group compared to those who received standard fortification, although these conclusions are limited by the discontinuation of the study due to tolerance problems in the intervention group [39].

Mode of administration: The evidence recommends fortification with high protein content in liquid form, compared to those formulated with powdered BF fortifiers, mainly in terms of safety [72]. However, with respect to the comparison in terms of somatometry, the meta-analysis by Bridges et al. concluded that studies on fortification of BM with liquid whey hydrolysate and liquid casein hydrolysate reported better growth rates, although with a low level of evidence [50]. Another RCT with 158 infants with birthweight <1,500 g found no difference in weight, length, or head circumference, although there were differences in knee-heel length between the liquid fortifier versus powder fortifier groups [45]. Regarding the initiation of fortification, the current evidence does not provide guidance on the optimal timing of onset. Among the studies on the influence of early versus late fortification, little or no difference in growth was found according to the timing of fortification initiation [42,55,58]. In two MAs, early fortification did not show a difference in time to achieve full enteral feeding. However, due to the very low or low quality of the evidence, the conclusions are uncertain [55,65].

Fortification Strategies and Neurodevelopment

Another aspect regarding the optimization of fortification is its implication in neurodevelopment and thus to analyze what modifications in the fortification strategy could improve the neurological development of PTNs.

Moreira-Monteagudo et al. in their SR concluded that psychomotor development was not affected by the feeding method, although they highlighted worse neurodevelopment results in feeding with unfortified BM compared to fortification or artificial formula, probably due to the lack of nutrient supply [46].

In 2022, Klamer et al. conducted an RCT where they compared different neurodevelopmental variables such as verbal comprehension or IQ between a group of premature babies fed with exclusive breastfeeding without fortified breastfeeding and premature formula, from hospital discharge to four months of corrected age, with no differences between the breastfed groups but better results for both compared to feeding with premature formula. It concludes that breastfeeding with/without fortification is an adequate nutritional method after discharge, at least from the point of view of neurodevelopment [43].

Regarding the effect of protein supplementation on neurodevelopment (high protein intake of 4-5 g/kg/day versus moderate protein intake of 3.5-4 g/kg/day), Kashaki et al. analyzed the difference in neurocognitive development in different areas at three years among premature infants with standard fortification and standard fortification with added protein content and concluded that protein intake in low birthweight newborns could improve neurological development on communication and gross motor skills. The rest of parameters did not show statistically significant variability depending on the protein intake [69].

In terms of fortification method and its implication in neurodevelopment, Reis et al. compared adjusted + directed fortification versus adjusted fortification only and assessed its influence on neurodevelopment and reported no significant differences in Bayley scores between both groups [37]. However, it has already been indirectly reported that targeted fortification can translate into an increase in fat-free mass, a fact that has recently been established as an indicator of better neurological development in PTN [64].

Safety Analysis of Different Fortification Strategies: Impact on Gut Microbiota

Although benefits of human milk fortification are evident, its effects on gastrointestinal microbiota seem more controversial, as well as the relationship between microbiome obtained and the beneficial impact of human milk on PTN. Asbury et al. indicate that the differences in gut microbiota depend in part on nutrients’ origin (human vs bovine) and have a direct dose-dependent relationship with the different nutritional components (BM, DM, fortifier). Newborns fed with exclusive human feeding/fortification show a lower microbial diversity and more uniform microbial profiles characterized by a higher abundance of Proteobacteria and Enterobacteriaceae. This could also be explained by the homogenization process of the fortifier of human origin, which translates into less microbial diversity [40]. In this sense, another RCT agrees in highlighting the importance of source (mother's own milk vs donated milk, with a better microbiological profile in fed with own mother's milk group) in the type of microbiota over the bovine or human origin of the fortifier, which had a minimal impact [44]. SR by Moreira-Monteagudo et al. reported that the pasteurization process of donated milk has a multifactorial influence on microbial development, less similar to that of the breastfed term newborn [46]. Finally, Embleton et al. analyzed differences in the microbiota between two groups of PTN: one fed with an exclusive human nutrition (donated ± BM and fortifiers derived from human milk) and a second fed with BM ± formula and fortifiers of bovine origin, without appreciating differences in microbiota patterns, suggesting that the impact that HM may have on reduction of comorbidities does not appear to be related to the microbiome [35].

Security Analysis of Different Fortification Strategies: Impact on Comorbidities (NEC, Sepsis)

When comparing fortification with human milk derivatives with bovine fortification, Embleton et al., Nogueira-Pileggi et al., and Premkumar et al. found no statistically significant differences in the incidence of NEC and sepsis [35,47,70]. On the other hand, Grace et al.'s meta-analysis suggests that the incidence of NEC is lower in the group of preterm infants fed BF-derived fortifiers compared to the group fed bovine fortifiers, with no differences in sepsis, with a low level of evidence [52].

With respect to the incidence of comorbidities depending on the time of initiation of fortification, the problem arises that not all authors take the same cut-off points to consider early and late fortification. However, both the MAs and Alyahya et al.’s SR do not seem to show (with low evidence) differences in NEC and sepsis between early fortification and late fortification [42,55,65]. Only a meta-analysis published in 2020 found an increasing NEC in the early fortification group [58].

The SR by Moreira-Monteagudo et al. emphasizes the lower incidence of NEC and sepsis of breastfeeding when compared to the starter formula or the use of fortification (especially powder), probably due to the difference in handling in the preparation [46]. In relation to this, Bridges e tal. found no significant differences when comparing the fortification of BM using liquid versus powdered human milk derivatives [50]. In contrast, Masoli et al.'s CT with 158 preterm infants found, although not statistically significant, that incidence of NEC/death was higher in the group of fortification with powdered human milk derivatives versus fortification with liquid human milk derivatives (8.1% vs 1.3%) [45].

Likewise, the incidence of these comorbidities in relation to the amount of fortification proteins has also been analyzed. Hemmati and Ghassemzadeh compared extra protein fortification versus standard protein fortification, finding no cases of NEC in either group [36]. Gao et al. found no evidence that the amount of protein concentration in fortification modified the incidence of sepsis and NEC [62]. On the other hand, from Hamidi et al.'s study, it can be deduced that receiving protein supplements in high doses causes an increase in neutrophil and lymphocyte counts and serum IgA concentration, which could help improve immune system [41].

Discussion

Evidence supports fortification of human milk for PTN [5-7]. Exclusive breastfeeding, without fortification, appears to have a higher incidence of growth retardation and neurodevelopment [7,9,10,46] However, there is currently no consensus on the clinical management of enteral nutrition supplementation of PTNs.

According to the present review, high-dose protein supplementation (4-5 g/kg/day) significantly improves anthropometric parameters [36,41,48,64]. In addition, high-protein intake may improve neurological development in terms of communication and gross motor skills [69]. In terms of complications derived from increased protein intake, it has not been shown to modify the incidence of sepsis and NEC, and thus it could be a safe and effective strategy [41,62].

On the other hand, there is no evidence that protein source (human vs bovine fortification) influences body growth and composition [49], whereas HM-based fortification may reduce the incidence of NEC compared to infant formula or fortifying formula (especially powder) [52]. This is probably due to the difference in handling when preparing both the artificial milk bottles and the fortification itself, whether in liquid or powder form. Regarding changes in the microbiota, it seems that supplementation with nutrients from maternal origin in the early stages of life modifies microbial development, with more uniform profiles and less microbiological diversity, with no clear evidence of its relationship with an increase in enteral intolerance or NEC [40]. In fact, the influence of the type of human milk (BM vs donor) does have a greater influence on the gut microbiological profile than the origin of the fortifier [44], although there does not seem to be a clear relationship between the microbiome obtained through an exclusive human diet and the lower incidence of NEC or sepsis presented by PTNs fed in this way [35].

Regarding the fortification method, individualized fortification in its two variants improves somatometric results compared to standard fortification [68], while in individualized fortification, directed method had more positive effects on growth in the short term, as it manages to adjust protein intake depending on the macronutrients obtained in the milk itself, thus making an individualized adjustment for each PTN [60,61]. About neurodevelopment, it is not possible to establish clear differences that would allow one of the individualized fortification methods to be recommended [37].

Early versus late onset does not differentiate between better somatometric targets [42,55,58,65]. On the other hand, when comparing comorbidities according to the time of fortification initiation, there is no clear level of evidence, and not all studies took the same cut-off points to consider early and late fortification [42,65]. The studies analyzed, with a low level of evidence, show no differences regarding the development of comorbidities (sepsis and NEC) between early versus late fortification [42,55,65].

Comparing liquid versus powder fortification, updates in the evidence-based literature have led experts to recommend the intake of high-protein fortifiers in liquid form, compared to those formulated with powdered LM fortifiers, for the purposes of both the incidence of comorbidities and somatometric variables. However, low level of evidence does not allow definitive recommendations to be made [45,50].

## Conclusions

Fortification of human milk is a recommended nutritional strategy for PTNs. High protein intake (4-5 g/kg/day) in fortification seems to be beneficial for growth and neurodevelopment of these patients, without increasing the risk of comorbidities. Human milk-based fortification reduces the incidence of NEC, with no improvement in somatometric or neurodevelopmental parameters. Individualized fortification, especially “directed” modality, has shown better results over standard fortification. Future investigation strategies should compare different amounts of protein in multicomponent fortifiers and be designed to determine effects on length of hospital stay and safety, as well as on long-term growth, body composition, and cardiometabolic and neurodevelopmental outcomes. The application of standardized and homogeneous protocols in terms of initiation, nutritional balance, and method should be the objective for the optimization of fortification strategies.
